# Supplementary material for: Echovirus-30 Infection Alters Host Proteins in Lipid Rafts at the Cerebrospinal Fluid Barrier In Vitro
Source: Microorganisms. 2020 Dec 10;8(12):1958. doi: 10.3390/microorganisms8121958 (PMC7764136; doi:10.3390/microorganisms8121958)
Supplement: Supplementary file 1 [file microorganisms-08-01958-s001.pdf]

Supplemental data:

Supplemental figure 1. Infection efficacy, cytoxicity and decrease in barrier integrity of HIBCPP cells following infection with MOI 20 of E-30.

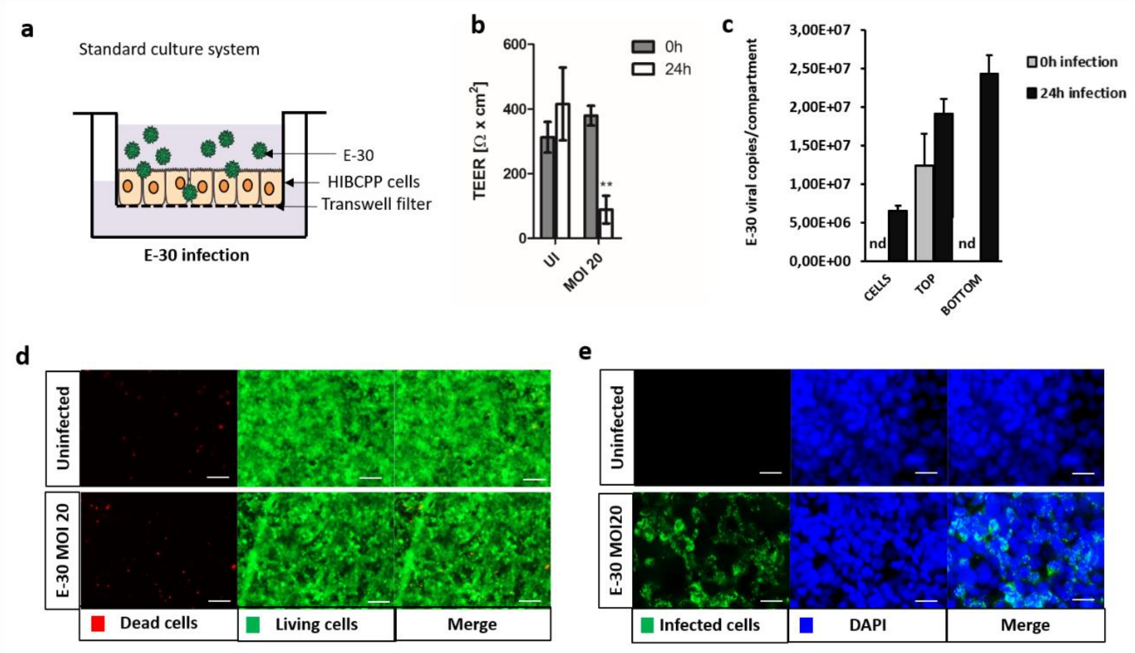

(a) Schematic representation of the culture model used for the experiments. (b) Barrier integrity of HIBCPP cell layers was evaluated following 24 h of infection with E-30 at MOI 20 by measuring the transepithelial electrical resistance (TEER). The same analysis was performed for uninfected (UI) control. (c) Quantification of the E-30 viral particles present in each compartment via qPCR after 24 h at T=0 h and T=24 h of infection with E-30 with MOI 20 (nd=non detected). (d) Pictures of Live/Dead viability assays; the living cells are stained in green (cell tracker green) and the dead cells in red (ethidium homodimer-1). Scale bars represent 100 µm. (e) Immunofluorescence imaging of HIBCPP cell layers following 24 h of infection with E-30. Nuclei are stained with DAPI (blue), HIBCPP cells infected with E-30 are stained with anti-PAN entero (green). Scale bars represent 15 µm. Data are shown as mean ± SD of 3 independent experiments, each of them performed in triplicates. For statistical analysis, Student's *t*-test was performed with the software Graphpad QuickCalcs. p values are displayed as follows: ** $p<0.001$.

Supplemental table 1. Up-regulated proteins in the lipid raft of HIBCPP cells following 24 h of E-30 infection at MOI 20. Proteins were identified as significantly changed in abundancy, when they exceeded a t-test difference > 1.0. Results shown are the comparison of 3 different independent proteomic experiments.

Protein names	Gene names	Sequence coverage [%]	Mol. weight [kDa]	Log2 (LFQ) control	Log2 (LFQ) E-30	Fold Change	-Log (pvalue)
Inner nuclear membrane protein Man1	LEMD3	>25	100,0	NaN	31,24	4,78	2,52
Probable dolichyl pyrophosphate Glc1Man9GlcNAc2 alpha-1,3	ALG8	>25	#N/A	NaN	29,81	3,27	1,96
Uncharacterized protein C19orf52	C19orf52	>25	#N/A	27,47	31,99	2,68	1,46
Neutral amino acid transporter B(0)	SLC1A5	>25	56,6	30,71	31,88	2,22	1,63
Mitochondrial thiamine pyrophosphate carrier	SLC25A19	>25	35,5	29,25	31,04	2,21	1,37
Inositol 1,4,5-trisphosphate receptor type 3	ITPR3	>25	304,1	33,38	34,86	2,17	1,43
Autophagy-related protein 9A	ATG9A	>25	94,4	26,54	30,21	2,15	1,17
ADP/ATP translocase 2	SLC25A5	>25	32,9	36,04	37,43	2,15	1,44
Endoplasmic reticulum-Golgi intermediate compartment protein	ERGIC1	>25	32,6	28,80	30,31	2,05	1,18
Glycerophosphodiester phosphodiesterase domain	GDPD3	>25	36,6	28,49	29,70	1,99	1,30
Solute carrier family 25 member 40	SLC25A40	>25	38,1	28,66	30,07	1,99	1,20
Mitochondrial dicarboxylate carrier	SLC25A10	>25	31,3	32,92	34,62	1,94	1,18
Niemann-Pick C1 protein	NPC1	>25	142,2	29,85	31,14	1,93	1,29
Corticosteroid 11-beta-dehydrogenase isozyme 2	HSD11B2	>25	44,1	28,54	30,50	1,93	1,13
Rabankyrin-5	ANKFY1	>25	128,4	29,83	31,50	1,91	1,16
Delta(14)-sterol reductase	TM7SF2	>25	30,4	30,19	30,70	1,89	1,05
Mitochondrial glutamate carrier 1	SLC25A22	>25	34,5	30,62	32,22	1,89	1,16
Golgi resident protein GCP60	ACBD3	>25	60,6	27,64	29,89	1,88	1,06
Delta(24)-sterol reductase	DHCR24	>25	#N/A	30,69	31,65	1,88	1,44
ADP/ATP translocase 1	SLC25A4	>25	33,1	30,51	32,14	1,86	1,13
ATP synthase subunit f, mitochondrial	ATP5J2	>25	10,9	30,45	31,88	1,81	1,14
Phosphate carrier protein, mitochondrial	SLC25A3	>25	36,2	34,81	36,31	1,79	1,11
Transmembrane protein 33	TMEM33	>25	28,0	31,81	33,19	1,78	1,13
7-dehydrocholesterol reductase	DHCR7	>25	54,5	33,08	34,43	1,77	1,13
Ras-related protein Rap-1b	RAP1B	>25	20,8	32,32	33,04	1,76	1,57
Carcinoembryonic antigen-related cell adhesion molecule 1	CEACAM1	>25	57,6	30,48	31,52	1,76	1,25
Prohibitin	PHB	>25	29,8	36,45	37,72	1,74	1,12
Lysophospholipid acyltransferase 5	LPCAT3	>25	56,0	29,57	30,82	1,73	0,95
Prohibitin-2	PHB2	>25	33,3	36,34	37,69	1,71	1,08
ADP/ATP translocase 3	SLC25A6	>25	32,9	33,12	34,13	1,62	1,12
Cation-dependent mannose-6-phosphate receptor	M6PR	>25	31,0	30,08	30,73	1,61	1,50
Transmembrane 9 superfamily member 1	TM9SF1	>25	92,4	29,88	31,12	1,61	0,91
Canalicular multispecific organic anion transporter 2	ABCC3	>25	169,3	31,28	32,11	1,61	1,24
Derlin-2	DERL2	>25	#N/A	NaN	29,29	1,60	1,11
Glucose-6-phosphate translocase	SLC37A4	>25	46,4	29,02	29,68	1,60	0,89
Aspartate--tRNA ligase, mitochondrial	DARS2	>25	73,6	29,13	29,26	1,57	0,89
H(+)/Cl(-) exchange transporter 3	CLCN3	>25	87,9	28,51	30,11	1,51	0,87
Anion exchange protein 2	SLC4A2	>25	137,0	31,45	32,37	1,51	1,06

Protein unc-45 homolog A	UNC45A	>25	118,4	29,32	30,04	1,50	1,21
GPI transamidase component PIG-T	PIGT	>25	66,0	32,45	33,66	1,49	0,93
Succinate dehydrogenase [ubiquinone] iron-sulfur subunit	SDHB	>25	31,6	30,86	33,12	1,48	0,78
Rhomboid domain-containing protein 2	RHBDD2	>25	39,2	29,93	30,73	1,46	1,08
Protein THEM6	THEM6	>25	23,9	30,76	31,98	1,46	0,89
ER membrane protein complex subunit 4	EMC4	>25	7,8	29,51	30,62	1,44	0,90
28S ribosomal protein S34, mitochondrial	MRPS34	>25	26,3	31,02	30,64	1,41	0,75
Lysophosphatidylcholine acyltransferase 2	LPCAT2	>25	60,2	30,32	30,90	1,41	0,85
Carnitine O-palmitoyltransferase 1, liver isoform	CPT1A	>25	88,4	33,09	34,16	1,41	0,89
Polypeptide N-acetylgalactosaminyltransferase 3	GALNT3	>25	72,6	31,05	31,71	1,40	1,15
Sodium- and chloride-dependent neutral	SLC6A14	>25	72,2	29,43	30,56	1,40	0,86
Succinate dehydrogenase [ubiquinone] flavoprotein subunit	SDHA	>25	72,7	32,56	34,24	1,39	0,76
Methyltransferase-like protein 7B	METTL7B	>25	31,3	30,91	31,84	1,38	0,92
Dolichyl-diphosphooligosaccharide--protein glycosyltransferase	STT3B	>25	93,7	34,32	35,61	1,38	0,81
Transmembrane 9 superfamily member 3	TM9SF3	>25	67,9	33,04	33,97	1,37	0,90
Polyribonucleotide nucleotidyltransferase 1, mitochondrial	PNPT1	>25	#N/A	27,80	29,90	1,36	0,72
Protein canopy homolog 2	CNPY2	>25	29,2	29,22	30,37	1,36	0,82
GPI-anchor transamidase	PIGK	>25	45,3	31,68	33,21	1,35	0,75
2-hydroxyacylsphingosine 1-beta-galactosyltransferase	UGT8	>25	61,4	28,74	29,42	1,34	0,78
Thioredoxin-dependent peroxide reductase, mitochondrial	PRDX3	>25	27,7	30,80	32,92	1,34	0,70
Fermitin family homolog 1	FERMT1	>25	77,4	28,41	28,87	1,33	0,95
Mitochondrial inner membrane protein OXA1L	OXA1L	>25	45,2	30,24	31,35	1,32	0,80
NAD(P) transhydrogenase, mitochondrial	NNT	>25	113,9	33,19	35,20	1,32	0,69
Acyl-CoA dehydrogenase family member 9, mitochondrial	ACAD9	>25	68,8	32,09	33,25	1,31	0,78
Piezo-type mechanosensitive ion channel component 1	PIEZO1	>25	286,8	30,78	31,51	1,31	0,76
Rho-related GTP-binding protein RhoC	RHOC	>25	21,5	32,77	33,34	1,31	1,13
Mitochondrial ornithine transporter 1	SLC25A15	>25	32,7	29,22	31,11	1,30	0,69
Heat-stable enterotoxin receptor	GUCY2C	>25	#N/A	27,46	30,10	1,30	0,69
Protein sel-1 homolog 1	SEL1L	>25	88,8	30,15	31,87	1,29	0,69
Phosphatidylinositide phosphatase SAC1	SACM1L	>25	67,0	30,92	31,59	1,28	0,97
Ras-related protein Rab-10	RAB10	>25	22,5	32,63	33,19	1,28	1,10
FAS-associated factor 2	FAF2	>25	52,6	32,88	33,59	1,27	0,93
B-cell receptor-associated protein 31	BCAP31	>25	28,0	33,41	34,15	1,27	0,91
Urokinase plasminogen activator surface receptor	PLAUR	>25	#N/A	28,99	29,41	1,27	0,99
2-5-oligoadenylate synthase 2	OAS2	>25	83,2	34,48	35,05	1,27	1,08
39S ribosomal protein L28, mitochondrial	MRPL28	>25	30,2	30,70	30,35	1,27	0,65
Interferon-induced protein with tetratricopeptide repeats 1	IFIT1	>25	55,4	29,11	30,71	1,26	0,68
Peroxisomal membrane protein 11B	PEX11B	>25	28,4	29,55	30,35	1,26	0,69
NADH-cytochrome b5 reductase 3	CYB5R3	>25	34,2	33,85	34,58	1,25	0,89
Ethanolaminephosphotransferase 1	EPT1	>25	45,1	29,32	30,25	1,25	0,79
4F2 cell-surface antigen heavy chain	SLC3A2	>25	64,9	34,16	34,71	1,25	1,07
Nuclear autoantigen Sp-100	SP100	>25	100,4	29,67	29,59	1,25	0,70
Calcium-binding mitochondrial carrier protein Aralar2	SLC25A13	>25	74,2	34,65	35,73	1,25	0,75

Calcium-binding mitochondrial carrier protein Aralar1	SLC25A12	>25	74,8	32,64	33,90	1,23	0,70
Minor histocompatibility antigen H13	HM13	>25	41,5	31,47	32,99	1,23	0,66
Vacuole membrane protein 1	VMP1	>25	46,2	29,50	30,46	1,22	0,75
Anoctamin-6	ANO6	>25	#N/A	29,34	31,28	1,22	0,60
Elongation factor Tu, mitochondrial	TUFM	>25	49,5	34,05	34,70	1,21	0,90
Cleft lip and palate transmembrane protein 1	CLPTM1	>25	76,1	29,18	30,38	1,21	0,69
Major facilitator superfamily domain-containing protein 10	MFSD10	>25	#N/A	30,73	30,23	1,20	0,61
ADP-dependent glucokinase	ADPGK	>25	54,1	29,18	29,92	1,19	0,64
Transmembrane protein 87A	TMEM87A	>25	63,4	29,76	31,12	1,18	0,65
AP-1 complex subunit beta-1	AP1B1	>25	104,6	29,13	29,84	1,18	0,81
Epoxide hydrolase 1	EPHX1	>25	52,9	32,67	33,26	1,18	0,91
39S ribosomal protein L19, mitochondrial	MRPL19	>25	33,5	31,19	30,40	1,18	0,60
Sideroflexin-3	SFXN3	>25	35,5	32,38	33,54	1,18	0,67
Mitochondrial import inner membrane translocase subunit TIM50	TIMM50	>25	39,6	31,52	32,49	1,17	0,71
Complex I assembly factor TIMMDC1, mitochondrial	TIMMDC1	>25	32,2	29,71	31,40	1,16	0,60
Sarcoplasmic/endoplasmic reticulum calcium ATPase 2	ATP2A2	>25	114,8	37,02	37,71	1,16	0,80
Uridine 5-monophosphate synthase	UMPS	>25	52,2	28,83	29,25	1,16	0,69
Dolichyl-diphosphooligosaccharide--protein glycosyltransferase 2	RPN2	>25	69,3	36,22	37,02	1,15	0,74
Ceramide synthase 6	CERS6	>25	44,9	31,20	32,05	1,15	0,72
Transmembrane channel-like protein 4	TMC4	>25	78,8	29,09	29,46	1,14	0,82
28S ribosomal protein S2, mitochondrial	MRPS2	>25	33,2	30,80	30,44	1,14	0,62
Extended synaptotagmin-2	ESYT2	>25	98,0	28,89	29,57	1,13	0,79
Translocating chain-associated membrane protein 1	TRAM1	>25	33,4	32,47	33,25	1,11	0,70
Lipoamide acyltransferase	DBT	>25	53,5	30,29	29,66	1,11	0,58
Endothelin-converting enzyme 1	ECE1	>25	83,6	28,11	28,38	1,10	0,76
Glucosylceramidase	GBA	>25	50,3	28,82	28,82	1,10	0,66
Endoplasmic reticulum lectin 1	ERLEC1	>25	54,9	28,88	30,08	1,09	0,57
ATP synthase subunit delta, mitochondrial	ATP5D	>25	17,5	30,88	32,02	1,09	0,60
39S ribosomal protein L47, mitochondrial	MRPL47	>25	29,5	29,28	30,68	1,09	0,55
Lysocardiolipin acyltransferase 1	LCLAT1	>25	48,9	30,39	31,73	1,08	0,57
Interferon-induced protein with tetratricopeptide repeats 3	IFIT3	>25	56,0	28,74	29,37	1,08	0,75
Long-chain fatty acid transport protein 4	SLC27A4	>25	72,1	28,74	30,16	1,07	0,56
Mitochondrial 2-oxoglutarate/malate carrier protein	SLC25A11	>25	34,1	33,28	34,55	1,07	0,57
28S ribosomal protein S30, mitochondrial	MRPS30	>25	50,4	29,94	29,93	1,07	0,54
ATP synthase subunit g, mitochondrial	ATP5L	>25	8,5	31,18	32,31	1,06	0,58
28S ribosomal protein S25, mitochondrial	MRPS25	>25	20,1	31,72	30,49	1,06	0,52
Antigen peptide transporter 2	TAP2	>25	77,6	33,55	34,78	1,05	0,56
39S ribosomal protein L37, mitochondrial	MRPL37	>25	48,1	32,20	31,29	1,05	0,51
39S ribosomal protein L10, mitochondrial	MRPL10	>25	29,3	28,81	29,51	1,05	0,57
Protein S100-A6	S100A6	>25	9,7	34,80	35,23	1,05	0,97
Mitochondrial import inner membrane translocase subunit Tim23	TIMM23	>25	21,9	29,83	31,15	1,05	0,55
Protein RER1	RER1	>25	23,0	32,12	33,08	1,05	0,60
Elongation of very long chain fatty acids protein 1	ELOVL1	>25	32,7	29,11	28,77	1,05	0,59

Cytochrome c oxidase subunit NDUFA4	NDUFA4	>25	9,4	29,74	30,86	1,05	0,58
Ras-related protein Ral-A	RALA	>25	23,6	30,78	31,80	1,05	0,59
Monoacylglycerol lipase ABHD12	ABHD12	>25	45,1	29,67	31,19	1,05	0,53
Adenosine 3-phospho 5-phosphosulfate transporter 1	SLC35B2	>25	47,5	31,51	32,16	1,04	0,70
Magnesium transporter protein 1	MAGT1	>25	41,5	33,43	34,38	1,04	0,60
Protein ERGIC-53	LMAN1	>25	57,5	34,44	35,34	1,03	0,60
ATP synthase subunit beta, mitochondrial	ATP5B	>25	56,6	36,41	37,03	1,03	0,71
39S ribosomal protein L2, mitochondrial	MRPL2	>25	24,4	30,38	29,72	1,03	0,53
Integral membrane protein GPR180	GPR180	>25	49,4	28,29	29,22	1,03	0,59
Acyl-CoA:lysophosphatidylglycerol acyltransferase 1	LPGAT1	>25	43,1	29,18	30,10	1,02	0,59
GPI transamidase component PIG-S	PIGS	>25	61,7	32,68	33,87	1,02	0,54
Diablo homolog, mitochondrial	DIABLO	>25	#N/A	30,22	31,10	1,02	0,51
ATP synthase subunit alpha, mitochondrial	ATP5A1	>25	59,8	36,49	37,09	1,02	0,70
Dipeptidase 1	DPEP1	>25	45,7	29,10	29,36	1,02	0,53
Acetylcholinesterase	ACHE	>25	67,8	28,29	28,71	1,01	0,69
Sarcolemmal membrane-associated protein	SLMAP	>25	95,2	29,64	30,93	1,01	0,53
Acetoacetyl-CoA synthetase	AACS	>25	75,1	28,98	29,31	1,01	0,52
ATP synthase subunit O, mitochondrial	ATP5O	>25	23,3	33,69	34,59	1,00	0,57

Supplemental table 2. Downregulated proteins in the lipid raft of HIBCPP cells following 24 h of E-30 infection at MOI 20. Proteins were identified as significantly changed in abundancy, when they exceeded a t-test difference < -1.0. Results shown are the comparison of 3 different independent proteomic experiments.

Protein names	Gene names	Sequence coverage [%]	Mol. weight [kDa]	Log 2 (LFQ) control	Log 2(LFQ) E-30	Fold Change	-Log (pvalue)
Major vault protein	MVP	>25	99,3	36,49	35,09	-3,16	2,34
Tumor protein D52	TPD52	>25	31,2	30,01	28,43	-2,88	1,89
Radixin	RDX	>25	68,6	30,76	29,30	-2,28	1,53
Dynein light chain roadblock-type 1	DYNLRB1	>25	10,9	29,18	28,47	-2,21	2,06
Splicing factor U2AF 35 kDa subunit	U2AF1	>25	27,9	30,77	30,06	-2,04	2,08
Perilipin-3	PLIN3	>25	47,1	31,09	28,76	-1,99	1,13
Poly [ADP-ribose] polymerase 4	PARP4	>25	192,6	30,41	28,50	-1,96	1,10
Splicing factor 3A subunit 3	SF3A3	>25	58,8	29,64	28,68	-1,93	1,31
Prefoldin subunit 6	PFDN6	>25	14,6	28,89	NaN	-1,84	1,24
WD repeat-containing protein 61	WDR61	>25	33,6	28,61	28,24	-1,77	1,12
26S proteasome non-ATPase regulatory subunit 9	PSMD9	>25	24,7	29,95	29,20	-1,75	1,05
Nuclear protein localization protein 4 homolog	NPLOC4	>25	68,1	29,60	28,67	-1,70	1,07
Histone H2A.V	H2AFV	>25	13,5	31,48	29,77	-1,68	0,98
Histone H1.0	H1F0	>25	20,9	30,01	28,55	-1,67	1,02
Palmitoyl-protein thioesterase 1	PPT1	>25	22,9	29,36	28,69	-1,67	1,11
Small nuclear ribonucleoprotein G	SNRPG	>25	8,5	30,66	29,57	-1,66	1,12
Dynein light chain 1, cytoplasmic	DYNLL1	>25	10,4	32,55	31,58	-1,63	1,15

NHP2-like protein 1	NHP2L1	>25	14,6	29,15	28,14	-1,62	0,97
Protein BRICK1	BRK1	>25	8,7	28,50	27,92	-1,60	1,62
Splicing factor 3B subunit 4	SF3B4	>25	44,4	29,88	29,29	-1,60	0,92
Lamina-associated polypeptide 2, isoform alpha	TMPO	>25	75,5	31,45	29,98	-1,53	0,90
Clathrin light chain A	CLTA	>25	27,1	32,72	31,94	-1,45	1,08
Tumor protein D54	TPD52L2	>25	23,8	31,08	29,85	-1,42	0,86
Nuclear pore complex protein Nup93	NUP93	>25	99,6	30,12	29,11	-1,41	0,92
Small nuclear ribonucleoprotein F	SNRPF	>25	9,7	29,62	28,82	-1,40	0,92
Splicing factor 3B subunit 5	SF3B5	>25	10,1	29,46	29,15	-1,39	0,85
Moesin	MSN	>25	67,8	32,12	31,09	-1,37	0,87
Src substrate cortactin	CTTN	>25	61,6	33,64	32,04	-1,36	0,75
U1 small nuclear ribonucleoprotein A	SNRPA	>25	31,3	31,20	31,80	-1,36	0,70
Chromodomain-helicase-DNA-binding protein 4	CHD4	>25	217,1	29,06	27,54	-1,35	0,75
Arfaptin-1	ARFIP1	>25	41,7	29,26	28,81	-1,34	0,87
Nuclear mitotic apparatus protein 1	NUMA1	>25	238,3	31,85	28,15	-1,34	0,70
Receptor expression-enhancing protein 5	REEP5	>25	21,5	28,73	28,53	-1,31	1,12
Tight junction protein ZO-3	TJP3	>25	#N/A	29,70	28,97	-1,31	0,93
Nesprin-2	SYNE2	>25	#N/A	35,29	33,92	-1,29	0,73
Histone H2A type 1-J	HIST1H2AJ	>25	18,5	30,78	29,78	-1,29	0,68
Small nuclear ribonucleoprotein-associated protein N	SNRPN	>25	17,5	29,76	29,28	-1,28	1,01
Apoptosis regulator BAX	BAX	>25	21,2	34,24	33,56	-1,25	0,91
Peptidyl-prolyl cis-trans isomerase A	PPIA	>25	18,0	36,12	35,35	-1,23	0,84
Spectrin alpha chain, non-erythrocytic 1	SPTAN1	>25	284,5	31,43	29,38	-1,23	0,63
Small nuclear ribonucleoprotein Sm D2	SNRPD2	>25	13,5	29,85	29,46	-1,23	0,74
Transcription factor BTF3	BTF3	>25	22,2	30,00	28,61	-1,23	0,65
26S proteasome non-ATPase regulatory subunit 4	PSMD4	>25	40,7	29,85	28,90	-1,20	0,72
Twinfilin-1	TWF1	>25	40,3	30,30	29,88	-1,18	0,64
Mitogen-activated protein kinase kinase kinase kinase 4	MAP4K4	>25	#N/A	28,94	29,17	-1,18	0,66
mRNA export factor	RAE1	>25	#N/A	32,66	32,16	-1,16	1,01
DnaJ homolog subfamily A member 1	DNAJA1	>25	44,9	30,60	29,77	-1,15	0,73
Nucleolar protein 58	NOP58	>25	59,6	30,56	31,04	-1,14	0,62
Pre-mRNA-processing factor 19	PRPF19	>25	55,2	30,49	29,93	-1,14	0,89
Large proline-rich protein BAG6	BAG6	>25	#N/A	33,93	33,14	-1,14	0,73
Actin-related protein 2	ACTR2	>25	44,8	30,56	31,02	-1,13	0,59
Histone-binding protein RBBP4	RBBP4	>25	47,7	31,07	30,35	-1,13	0,56
60S ribosomal protein L38	RPL38	>25	#N/A	30,76	29,54	-1,12	0,56
Thyroid hormone receptor-associated protein 3	THRAP3	>25	108,7	29,83	29,18	-1,12	0,61
Transcription factor BTF3 homolog 4	BTF3L4	>25	15,8	29,60	29,16	-1,11	0,78
AP-2 complex subunit alpha-2	AP2A2	>25	104,0	32,64	31,90	-1,11	0,72
Actin-related protein 2/3 complex subunit 5	ARPC5	>25	16,3	31,07	29,94	-1,11	0,61
Actin-related protein 2/3 complex subunit 1A	ARPC1A	>25	#N/A	30,26	29,69	-1,10	0,57
Macrophage migration inhibitory factor	MIF	>25	12,5	31,32	30,51	-1,10	0,68
Nascent polypeptide-associated complex subunit alpha	NACA	>25	15,0	30,03	28,58	-1,10	0,54

U2 small nuclear ribonucleoprotein A	SNRPA1	>25	28,4	35,85	35,20	-1,10	0,76
Spectrin beta chain, non-erythrocytic 1	SPTBN1	>25	274,6	31,85	31,12	-1,10	0,71
Matrin-3	MATR3	>25	94,6	33,20	32,01	-1,09	0,60
Heterogeneous nuclear ribonucleoproteins C1/C2	HNRNPC	>25	32,2	30,95	29,90	-1,09	0,62
Casein kinase I isoform alpha	CSNK1A1	>25	38,9	34,15	33,23	-1,09	0,64
40S ribosomal protein S6	RPS6	>25	28,7	30,99	28,34	-1,08	0,51
Splicing factor 3B subunit 2	SF3B2	>25	100,2	32,52	31,49	-1,08	0,61
Unconventional myosin-Ie	MYO1E	>25	127,1	31,77	31,18	-1,08	0,77
Clathrin light chain B	CLTB	>25	25,2	34,46	33,85	-1,07	0,76
Tight junction protein ZO-1	TJP1	>25	187,9	29,54	28,07	-1,07	0,53
Actin-related protein 3	ACTR3	>25	47,4	30,61	29,76	-1,07	0,53
Bcl-2-associated transcription factor 1	BCLAF1	>25	100,4	28,88	28,00	-1,06	0,62
U5 small nuclear ribonucleoprotein 200 kDa helicase	SNRNP200	>25	244,5	32,73	31,68	-1,06	0,57
Guanine nucleotide-binding protein-like 3	GNL3	>25	62,0	31,70	29,80	-1,05	0,50
V-type proton ATPase subunit G 1	ATP6V1G1	>25	13,8	31,57	29,97	-1,03	0,51
Actin-related protein 2/3 complex subunit 5-like protein	ARPC5L	>25	16,9	30,50	29,26	-1,03	0,53
F-actin-capping protein subunit alpha-1	CAPZA1	>25	32,9	34,58	34,14	-1,01	0,87
Eukaryotic translation initiation factor 4 gamma 1	EIF4G1	>25	158,6	33,82	33,11	-1,01	0,63
Stomatin-like protein 2, mitochondrial	STOML2	>25	38,5	34,20	33,39	-1,01	0,60
Actin-related protein 2/3 complex subunit 4	ARPC4	>25	#N/A	31,23	30,21	-1,01	0,55
Actin-related protein 2/3 complex subunit 2	ARPC2	>25	34,3	6,25	7,55	-1,00	32,73
RNA-binding protein Raly	RALY	>25	24,7	5,50	8,15	-1,00	31,64
